# Technical Development of a New Meningococcal Conjugate Vaccine

**DOI:** 10.1093/cid/civ595

**Published:** 2015-11-09

**Authors:** Carl E. Frasch, Subhash V. Kapre, Che-Hung Lee, Jean-Marie Préaud

**Affiliations:** 1 Frasch Biologics Consulting, Martinsburg, West Virginia; 2 Serum Institute of India, Ltd, Pune; 3 Center for Biologics Evaluation and Research, US Food and Drug Administration, Bethesda, Maryland; 4 Meningitis Vaccine Project, PATH, Ferney-Voltaire, France

**Keywords:** meningococcal, conjugate vaccine, group A, MenAfriVac, African meningitis belt

## Abstract

***Background.*** Group A *Neisseria meningitidis* has been a major cause of bacterial meningitis in the sub-Saharan region of Africa in the meningitis belt. *Neisseria meningitidis* is an encapsulated pathogen, and antibodies against the capsular polysaccharide are protective. Polysaccharide–protein conjugate vaccines have proven to be highly effective against several different encapsulated bacterial pathogens. Purified polysaccharide vaccines have been used to control group A meningococcal (MenA) epidemics with minimal success.

***Methods.*** A monovalent MenA polysaccharide–tetanus toxoid conjugate was therefore developed. This vaccine was developed by scientists working with the Meningitis Vaccine Project, a partnership between PATH and the World Health Organization.

***Results.*** A high-efficiency conjugation method was developed in the Laboratory of Bacterial Polysaccharides in the Center for Biologics Evaluation and Research and transferred to the Serum Institute of India, Ltd, which then developed methods for purification of the group A polysaccharide and used its tetanus toxoid as the carrier protein to produce the now-licensed, highly effective MenAfriVac conjugate vaccine.

***Conclusions.*** Although many years of application of meningococcal polysaccharide vaccines have had minimal success in preventing meningococcal epidemics in the meningitis belt of Africa, our collaborative efforts to develop a MenA conjugate vaccine yielded a safe and highly effective vaccine.

*Neisseria meningitidis* (the meningococcus) is a particularly important cause of bacterial meningitis in children and adults because of its potential to cause epidemics. The relative importance of meningococcal disease as a public health threat varies greatly over time and geographic location, but the epidemic potential of meningococci confers a special public health concern whenever clinical cases of meningococcal disease occur.

Meningococci are divided into 12 different groups based upon the expression of chemically and serologically different capsular polysaccharides (PSs) [1]. Virtually all meningococcal disease is caused by groups A, B, C, X, Y, and W. The relative importance of each group varies with geographic region. Group A meningococcal disease is largely a problem in sub-Saharan Africa, whereas groups C and Y account for more than half of the meningococcal disease in the United States. Group B *N. meningitidis* causes up to 90% of meningococcal disease in some European countries, while groups X and W have caused small- and moderate-sized outbreaks in Africa [2, 3].

Humans are the only natural host of meningococci, and about 5%–10% of adults are asymptomatic meningococcal carriers. Data from sub-Saharan Africa prior to introduction of the MenA conjugate vaccine have shown endemic carriage rates of <1% for group A meningococci [4].

## NEED FOR A GROUP A MENINGOCOCCAL CONJUGATE VACCINE

Major African epidemics are associated with group A meningococci [5]. Mongolia, Nepal, and India have also reported MenA epidemics over the last 20 years, but the disease burden is much smaller compared with that in sub-Saharan Africa [6]. The African “meningitis belt,” with a population of approximately 450 million people, is a huge area stretching from Senegal in the west to Ethiopia in the east. It was first described in 1963 by Lapeyssonnie [7]. Meningitis epidemics characteristically occur in the hot, dry, and dusty season from January to May and promptly cease with the onset of the rains. Focal epidemics occurred nearly every year in 1 or more of the meningitis belt countries, and large outbreaks occurred every 8–12 years [7, 8]. These epidemic cycles likely reflect major changes in population immunity over time [8].

In major African epidemics, attack rates range from 100 to 800 per 100 000 population, but individual communities have reported rates as high as 1%, caused almost entirely by group A meningococci [5]. These high rates occurred despite using millions of doses of group A/C PS vaccine administered in reactive campaigns in response to outbreaks. A MenA epidemic often lasts <2 months, and reactive campaigns require getting the infecting strain identified, finding vaccine, and obtaining funding for vaccine purchase plus operational costs. This work takes time, and reactive campaigns are often mounted late or even after a meningococcal epidemic has ended.

In 1996–1997, West Africa experienced one of the largest recorded outbreaks of epidemic meningitis in history, with >180 000 cases and 20 000 deaths registered. From 1998 to 2010, >700 000 new cases of acute meningitis were reported to the World Health Organization [8]. The most affected countries included Burkina Faso, Nigeria, Chad, Ethiopia, and Niger; in 2002, the outbreaks occurring in Burkina Faso, Ethiopia, and Niger accounted for about 65% of the total cases reported in the African continent. In 2009, northern Nigeria reported >70 000 cases of MenA meningitis. Furthermore, the meningitis belt appears to be extending farther south. In 2004, >11 000 cases of acute meningitis were reported from the Democratic Republic of Congo, a country heretofore not considered part of the meningitis belt.

## MENINGOCOCCAL POLYSACCHARIDE AND CONJUGATE VACCINES

Meningococcal PSs, like most other bacterial PS vaccines, do not effectively stimulate the immune system in young children and are largely nonimmunogenic in infants. The exception is the MenA PS, which, for reasons not well understood, is immunogenic in infants as young as 6 months of age, primes for a boosted response, and is effective when used in infants and toddlers in a 2-dose immunization schedule [9]. Nonetheless, and despite the use of tens of millions of doses of group A PS vaccines in Africa, MenA epidemics have continued to occur.

The development and use of meningococcal PS and conjugate vaccines have been reviewed [10–12]. The present review will focus only on MenA conjugate vaccines.

Initial studies on production and optimization of MenA conjugates were reported 40 years ago by Beuvery et al [13] and Jennings and Lugowski [14], well before commercialization of the *Haemophilus influenzae* type b conjugates. They described 2 differing conjugation methods for chemically linking the group A PS to a protein carrier. The first approach used partially depolymerized PS that was activated by creation of terminal aldehyde groups through periodate oxidation [14]. The reactive aldehydes were then combined through reductive amination to free amino groups, mostly on lysines, on the carrier protein in the presence of sodium cyanoborohydride. By this method, activation occurred at one specific site on the PS. The second approach utilized the carbodiimide reaction to covalently link carboxylic groups in the high-molecular-weight PS to lysine amino groups on the carrier protein [13]. Activation sites using this second method were more random. While site-specific activation is attractive from a biochemical standpoint, the random activation may, on average, have less deleterious effects upon individual PS epitopes.

The MenA conjugates prepared by Beuvery et al [13] were used to immunize mice. The coupling method using a 6-carbon spacer proved to be much more immunogenic than his method that used cyanogen bromide at pH 11 to create a cyanate group on the PS for coupling to the protein. It is likely that the high pH resulted in partial de–O-acetylation of the group A PS. Studies have clearly shown that the O-acetyl groups on the group A PS are critical for immunogenicity [15]. The group A conjugate stimulated T-cell–dependent responses because later immunization with the PS alone induced a boosted response. Beuvery also showed that the amount of free PS in a group C conjugate should be minimized (to about 10%), but does not need to be eliminated [16].

A monovalent MenA conjugate vaccine was developed for use in Africa because (1) the epidemiology of meningococcal disease in sub-Saharan Africa indicated that an effective monovalent MenA vaccine could prevent >90% of endemic and epidemic meningococcal disease, and (2) would be less expensive to develop than a polyvalent product.

Infants respond best to PS–protein conjugate vaccines because the immune response is more durable. Methods that have been used to prepare several meningococcal conjugate vaccines are now described [12, 17]. Currently licensed conjugate vaccines contain PS–protein hybrids formed by the covalent attachment of a protein through its amino acid groups to a chemically modified, or “activated” PS. Attachment of the protein provides a number of T-cell epitopes. These T-cell epitopes interact with CD4 helper T cells, greatly facilitating an antibody response to the attached PS. The T-cell–dependent response to a conjugate results in both serum immunoglobulin G (IgG) antibodies and memory B cells, even in infants. In general, immunogenicity of a PS–protein conjugate, in contrast to the native PS, does not depend on the size of the conjugated PS; conjugates prepared with either PS or oligosaccharides may have similar immunogenicity. The size requirement for highly immunogenic group A meningococcal conjugates has yet to be clearly demonstrated.

## DEVELOPMENT OF THE GROUP A CONJUGATE VACCINE FOR AFRICA

African public health officials from affected West African countries indicated at the time that to be affordable, the cost of a new meningococcal conjugate vaccine would need to be low (ie, US$0.50 per dose) [18]. At this price, no large pharmaceutical manufacturer was interested in developing a monovalent MenA conjugate vaccine for use in sub-Saharan Africa.

The Meningitis Vaccine Project (MVP) was established in 2001 with the goal to eliminate epidemic meningitis in Africa as a public health problem through the development of a MenA conjugate vaccine in partnership with a developing-country manufacturer (Serum Institute of India, Ltd [SIIL], in Pune) [19, 20]. The MVP staff identified an initial source of vaccine-grade MenA PS (SynCo Bio Partners B.V., Amsterdam, the Netherlands), a source of tetanus toxoid (SIIL), a heat-stable formulation (Aérial, Strasbourg, France), and a high-efficiency conjugation technology developed in the Laboratory of Bacterial Polysaccharides, Center for Biologics Evaluation and Research, US Food and Drug Administration. Technology transfers have been successfully performed at laboratory scale or pilot scale from 2004 to 2006. Production of the PS, purification, and conjugation processes were scaled up at SIIL and later shifted to a new building dedicated to the production of conjugate vaccines and fill finish at industrial scale. The initial MVP target was to be able to produce 25 million doses per year by 2009 [19], then to attain >50 million doses by 2012. In parallel, analytical methods for the evaluation of raw materials, conjugate bulk, and final product have been developed and fully validated prior to manufacturing clinical material. A stability program has been developed and demonstrated a very high stability of the material at all stages of production.

The challenges in the scale-up were considerable, especially to achieve uniform quality of the product. Analysis of 2-year production data at SIIL shows that the yields of all steps, free polysaccharide levels, and protein polysaccharide ratio were extremely satisfactory, showing the establishment of a large-scale process from the technology developed at laboratory scale. Thus, technology transfer from laboratory scale to pilot scale to full manufacturing was successfully completed.

## GROUP A CONJUGATE VACCINE PRODUCTION AND QUALITY CONTROL

For a PS to be chemically linked to a protein, the PS must be activated, that is, chemically modified. The 2 primary methods currently used for PS activation are periodate oxidation and cyanylation [21, 22]. Sodium periodate oxidizes diols (2 adjacent carbons with hydroxyl groups) into aldehydes (C = O) and in the process breaks C − C bonds. Thus, depending on the PS structure, periodate activation can fragment a PS and open the ring structures of sugars, thus altering PS conformation. In the case of the group A PS, only those repeat units lacking an O-acetyl group on carbon 3 can be activated by the periodate treatment, which is used in production of MenAfriVac. The percentage of O-acetylation in the purified group A PS is about 77%–85%, and O-acetylation is required for expression of protective epitopes on the PS [15].

**Figure 1.**
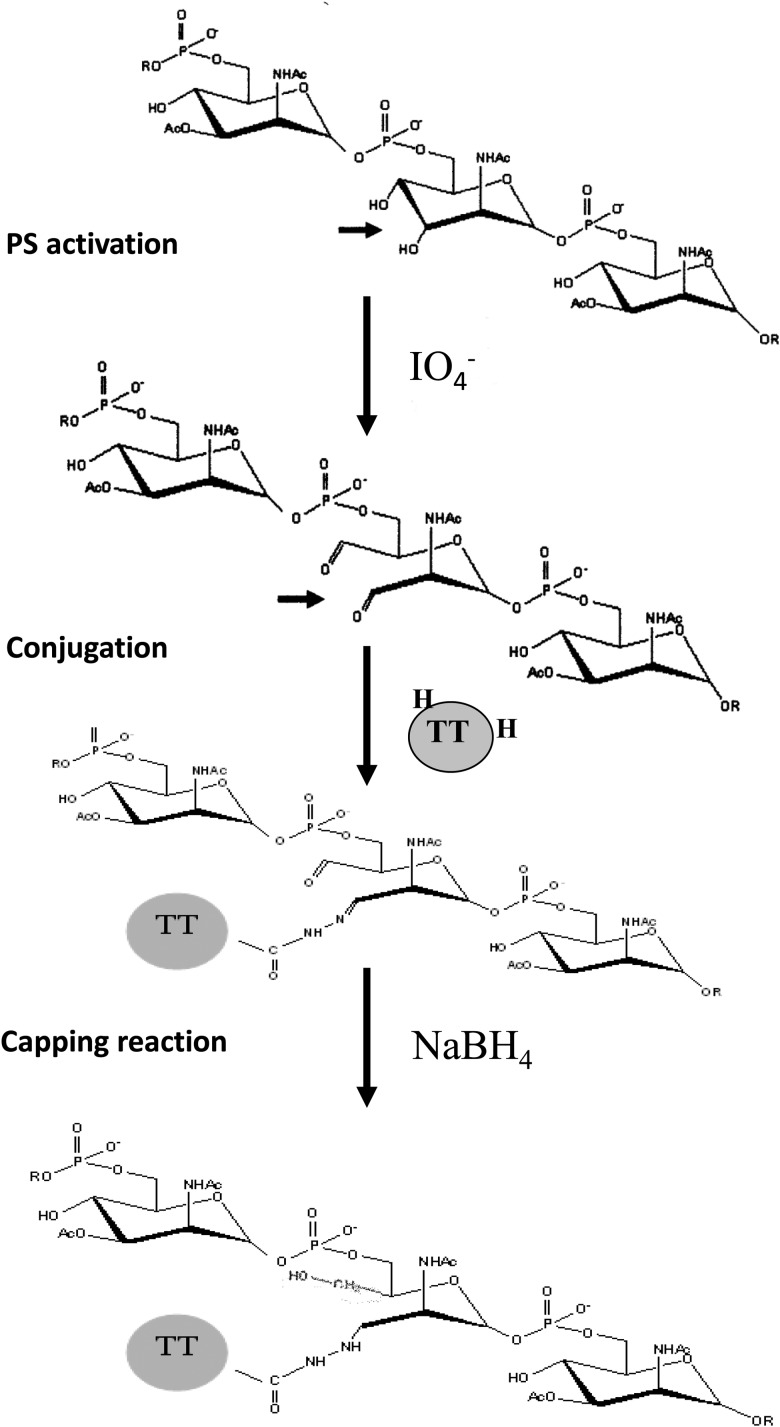
The group A meningococcal polysaccharide (PS) and schematics of polysaccharide activation and protein–polysaccharide conjugation. Abbreviation: TT, tetanus toxoid.

For most conjugates, the reactive aldehyde groups on the activated PS are condensed with free amino groups on the protein in the presence of sodium cyanoborohydride to form a stable secondary amine. Condensation of the aldehyde groups with the epsilon amino groups on lysine is a slow process, often taking a few days, with low conjugate yields. A new conjugation method was developed to decrease the conjugation time and increase conjugate yields (Figure 1) [23, 24].

Conjugation efficiency was improved for PsA-TT by chemically activating both the PS and carrier protein [23, 25]. Activated group A PS was reacted with either tetanus toxoid (TT) or hydrazide activated tetanus toxoid (TT-H) overnight (15–16 hours) at room temperature. The amount of high-molecular-weight conjugate obtained was much greater when the activated PS was mixed with TT-H compared with TT [23].

**Figure 2.**
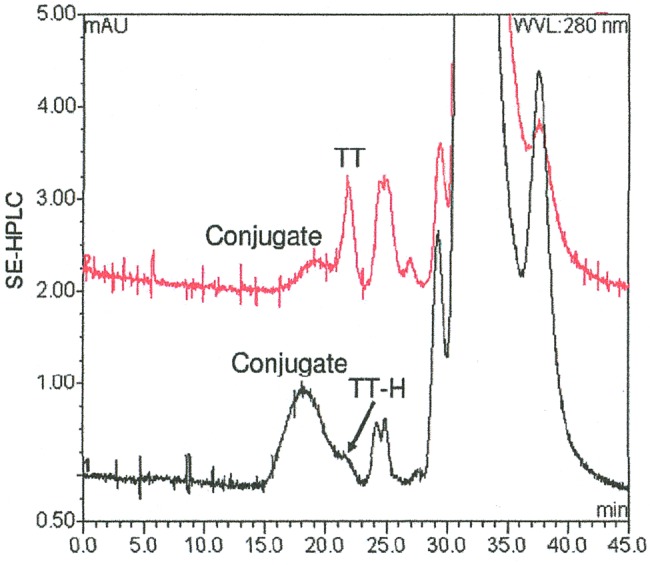
Improved conjugation efficiency as seen using size-exclusion high-performance liquid chromatography (SE-HPLC) when conjugates are prepared using hydrazide-activated tetanus toxoid (TT-H) compared with tetanus toxoid (TT).

A large number of lots of group A PS-TT conjugate vaccine were prepared at 1–2-mg, 25-mg, and 500-mg scales to ascertain the reproducibility and scalability of the conjugation method. As shown in Figure 1, the carboxyl groups of TT were first substituted with hydrazine in the presence of 1-ethyl-3(dimethylaminopropyl) carbodiimide under acidic conditions. The group A PS was activated by limited oxidation at the C3–C4 bond of the partially (5%–30%) de–O-acetylated polysaccharide with sodium periodate (Figure 1). The geometric mean degree of activation for TT is about 50 hydrazide groups per TT molecule, whereas that of the activated group A PS is about 100 saccharide repeats per aldehyde group [26]. The HPSEC profiles by a Waters ultrahydrogel linear column monitored at 206 nm of native and activated TT and group A PS, and the group A PS-TT conjugate, indicated that upon activation, the size of activated TT was unchanged from the native TT with an elution time of 22 minutes, suggesting that little aggregation occurred (Figure 2). The activated group A PS had a lower molecular weight than the native PS, suggesting some degree of degradation. After conjugation and subsequent reduction, a higher-molecular-weight peak appeared, indicating the formation of group A PS-TT conjugates (Figure 2), with much more conjugate produced when TT-H was used compared with TT.

Physicochemical analyses are needed at different steps in the manufacturing process, and there are a variety of alternative methodologies. Studies by Silveira et al in Brazil on a group C meningococcal conjugate using the TT-H technology showed that nuclear magnetic resonance could be used to show disappearance of the reactive aldehyde groups in the activated PS as a result of conjugation [27]. Additional advanced physicochemical methods for characterization of meningococcal conjugates have been described, including determination of hydrazine content [26, 28].

The described conjugation method produces high-molecular-weight cross-linked lattice structures, due to multiple aldehyde groups on the PS and multiple hydrazide groups on the TT. Similar conjugates have been produced using the group C and W meningococcal PSs, where they achieved 50% yields of conjugated PS [27, 29].

## GROUP A CONJUGATE IMMUNOGENICITY STUDIES IN ANIMALS

**Figure 3.**
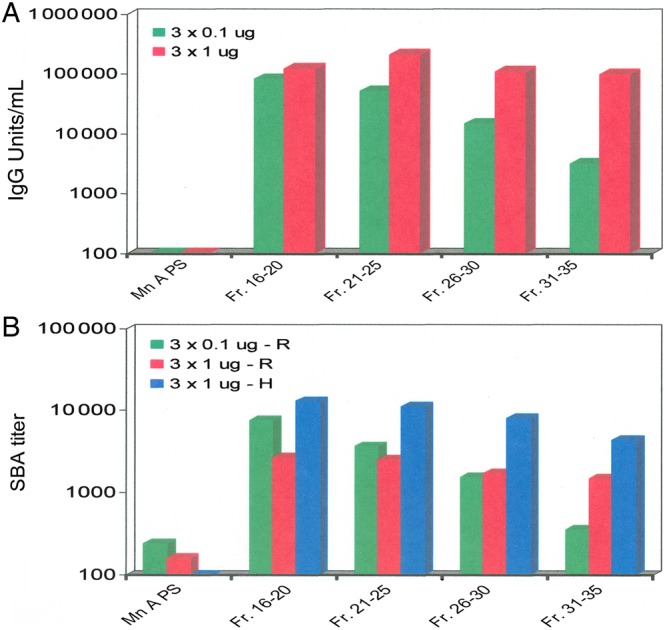
Immunogenicity of different molecular sizes of group A conjugate recovered from Sephacryl S-400 chromatography (see Figure 4) by enzyme-linked immunosorbent assay (ELISA) (*A*) and by serum bactericidal assay (SBA) (*B*). The ELISA antibody units were determined in comparison to a reference serum assigned 3200 units/mL. Geometric mean SBA titers are shown (*B*) for sera from 10 mice using rabbit (R) complement against group A strain F8238 and using human (H) complement using group A strain Z1092. The rabbit and human complement SBAs were performed in different laboratories [26]. Abbreviations: IgG, immunoglobulin G; Mn A PS, group A meningococcal polysaccharide.

Lots of the group A PS-TT conjugates were fractionated by S-400 size-exclusion chromatography to generate high- and low-molecular-weight conjugates. The S-400 fractions were combined according to molecular size into 4 pooled fractions (16–20, 21–25, 26–30, and 31–35) to study their immunogenicity in mice [23]. Regardless of differences in molecular size, all fractions induced high levels (≥100 000 units/mL) of anti–group A PS antibody in mice at the 3 × 1-µg PS dose compared with the native PS control group (<100 units/mL) (Figure 3*A*). The serum bactericidal antibody (SBA) titers of the antisera induced by all 4 fractions were rather high compared to the PS control (<160) when baby rabbit complement was used for the assay (Figure 3*B*). The SBA titers assayed with human complement showed similar trend as those obtained with baby rabbit complement, but with higher values (Figure 3*B*). This might be due to different bacterial strains, protocols, and other factors used in the assay methods carried out in 2 different laboratories. At 3 × 0.1-µg PS dose, the antibody levels induced by these fractions decreased with reduction of the conjugate size (Figure 3*A*), presumably due to less conjugated PS relative to total PS in the fractions of smaller molecular weight (fraction pool 31–35), as discussed below. SBA titers were similarly affected by conjugate size (Figure 3*B*).

**Figure 4.**
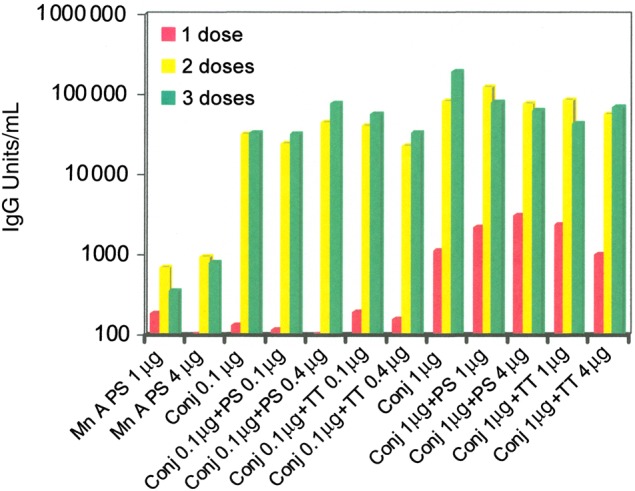
Geometric mean enzyme-linked immunosorbent assay (ELISA) antibody levels in sera from mice (n = 10) 2 weeks after 1, 2, or 3 immunizations every two weeks with group A polysaccharide (PS)–tetanus toxoid (TT) conjugate (Conj) lot MA031221J using different antigen concentrations and combinations as shown. ELISA units were determined in comparison with a reference serum assigned 3200 units/mL.

To test the quality and the potency of the conjugates, the immunogenicity of lot MA031221J was studied to evaluate the effect of lower dosages (0.1-µg PS dose) and the effect of 100%–400% added free PS or TT in the inoculum (Figure 4). Two weeks after the first injection, native group A PS and the conjugate groups at the 0.1-µg dose induced little antibody (≤200 units/mL), whereas the conjugate groups at the 1-µg dose induced noticeable amount of antibody (1000–3000 units/mL). Native group A PS displayed some immunogenicity (≤1000 unit/mL), whereas 0.1- and 1-µg doses of conjugate-induced much higher levels of antibody (≥22 000 units/mL) after the second or third injection. Presence of free PS or TT (100%–400%) had little effect on the potency of the conjugate at either the 0.1- or 1-µg PS dosage. The conjugate-induced antibody at the 0.1-µg PS dose was biologically functional as shown by SBA (data not presented); and the presence of free PS or TT (100%–400%), again, had little effect on the SBA titer of the induced response.

Finally, multiple lots of the MenA conjugate vaccine prepared at different manufacturing scales under current Good Manufacturing Practice conditions were characterized physicochemically and evaluated in mice and rabbits for both safety and immunogenicity prior to phase 1 trials in human adults [26].

## PSA-TT CONJUGATE VACCINE

The PsA-TT conjugate vaccine is a 10-dose lyophilized preparation that is reconstituted before injection. The vaccine is administered intramuscularly [30]. The vaccine was formulated to contain, per 0.5-mL dose, 10 µg of group A PS conjugated to 10–33 µg of TT, and 0.06 mg of Tris hydroxymethyl aminoethane when reconstituted with saline containing 0.01% thimerosal and 0.3 mg Al^+++^ as aluminum phosphate per dose. A 5-µg formulation has recently been prequalified by the World Health Organization for use in infants and toddlers in the Expanded Programme on Immunization.

## References

[1] Zollinger WD Meningococcal Meningitis. In: Cruz SJJ, ed. Vaccines and immunotherapy. New York: Pergamon Press, 1991:113–26.

[2] Boisier P, Nicolas P, Djibo S et al Meningococcal meningitis: unprecedented incidence of serogroup X-related cases in 2006 in Niger. Clin Infect Dis 2007; 44:657–63. 17278055 10.1086/511646

[3] Traoré Y, Njanpop-Lafourcade BM, Adjogble KL et al The rise and fall of epidemic *Neisseria meningitidis* serogroup W meningitis in Burkina Faso, 2002–2005. Clin Infect Dis 2006; 43:817–22. 16941360 10.1086/507339

[4] Kristiansen PA, Diomande F, Wei SC et al Baseline meningococcal carriage in Burkina Faso before the introduction of a meningococcal serogroup A conjugate vaccine. Clin Vaccine Immunol 2011; 18:435–43. 21228139 10.1128/CVI.00479-10 PMC3067389

[5] LaForce FM, Ravenscroft N, Djingarey M, Viviani S Epidemic meningitis due to group A *Neisseria meningitidis* in the African meningitis belt: a persistent problem with an imminent solution. Vaccine 2009; 27S:B13–9. 10.1016/j.vaccine.2009.04.062 19477559

[6] Sinclair D, Preziosi MP, John TJ, Greenwood B The epidemiology of meningococcal disease in India. Trop Med Int Health 2010; 15:1421–35. 21054695 10.1111/j.1365-3156.2010.02660.x

[7] Lapeyssonnie L La méningite cérébro-spinale en Afrique. Bull WHO 1963; 28:1–114. 14259333 PMC2554630

[8] LaForce FM, Okwo-Bele JM Eliminating epidemic group A meningococcal meningitis in Africa through a new vaccine. Health Aff (Millwood) 2011; 30:1049–57. 21653956 10.1377/hlthaff.2011.0328

[9] Lennon D, Gellin B, Hood D, Voss L, Heffernan H, Thakur S Successful intervention in a group A meningococcal outbreak in Auckland, New Zealand. Pediatr Infect Dis J 1992; 11:617–23. 1523071

[10] Frasch CE Meningococcal vaccines: past, present and future. In: Cartwright K, ed. Meningococcal disease. New York: Wiley, 1995:245–83.

[11] Zollinger WD New and improved vaccines against meningococcal disease. In: Levine MM, Woodrow GC, Kaper JB, Cobon GS, eds. New generation vaccines. New York: Marcel Dekker, 1997:469–88.

[12] Frasch CE, Bash MC *Neisseria meningitidis* vaccines. In: Ellis RW, Brodeur BR, eds. Bacterial vaccines. New York: Landes Bioscience, 2003:228–42.

[13] Beuvery EC, Kaaden AVD, Kanai V, Leussink AB Physiochemical and immunological characterization of meningococcal group A polysaccharide-tetanus toxoid conjugates prepared by two methods. Vaccine 1983; 1:31–6. 6442499 10.1016/0264-410x(83)90010-5

[14] Jennings HJ, Lugowski C Immunochemistry of groups A, B, and C meningococcal polysaccharide-tetanus toxoid conjugates. J Immunol 1981; 127:1011–8. 6790606

[15] Berry DS, Lynn F, Lee CH, Frasch CE, Bash MC Effect of O acetylation of *Neisseria meningitidis* serogroup A capsular polysaccharide on development of functional immune responses. Infect Immun 2002; 70:3707–13. 12065513 10.1128/IAI.70.7.3707-3713.2002 PMC128089

[16] Beuvery EC, Delft RV, Miedema F, Kanhai V, Nagel J Immunological evaluation of meningococcal group C polysaccharide-tetanus toxoid conjugate in mice. Infect Immun 1983; 41:609–17. 6409811 10.1128/iai.41.2.609-617.1983 PMC264686

[17] Frasch CE Preparation of bacterial polysaccharide-protein conjugates: analytical and manufacturing challenges. Vaccine 2009; 27:6468–70. 19555714 10.1016/j.vaccine.2009.06.013

[18] LaForce FM, Konde K, Viviani S, Preziosi MP The Meningitis Vaccine Project. Vaccine 2007; 25S:A97–100. 17521780 10.1016/j.vaccine.2007.04.049

[19] Bishai DM, Champion C, Steele ME, Thompson L Product development partnerships hit their stride: lessons from developing a meningitis vaccine for Africa*.* Health Aff 2011; 30:61058–64. 10.1377/hlthaff.2011.0295 21653957

[20] Jodar L, LaForce FM, Ceccarini C, Aguado T, Granoff DM Meningococcal conjugate vaccine for Africa: a model for development of new vaccines for the poorest countries. Lancet 2003; 361:1902–4. 12788589 10.1016/S0140-6736(03)13494-0

[21] Kniskern PJ, Marburg S Conjugation: design, chemistry, and analysis. In: Ellis RW, Granoff DM, eds. Development and clinical uses of *Haemophilus* b conjugate vaccines. New York: Marcel Dekker, 1994:37–69.

[22] Lees A, Nelson BL, Mond JJ Activation of soluble polysaccharides with 1-cyano-4-dimethylaminopyridinium tetrafluroborate for use in protein-polysaccharide conjugate vaccines and immunological reagents. Vaccine 1996; 14:190–8. 8920699 10.1016/0264-410x(95)00195-7

[23] Lee C-H, Kuo W-C, Beri S et al Preparation and characterization of an immunogenic meningococcal group A conjugate vaccine for use in Africa. Vaccine 2009; 27:726–32. 19063929 10.1016/j.vaccine.2008.11.065

[24] Frasch CE, Preziosi M-P, LaForce FM Development of a group A meningococcal conjugate vaccine MenAfriVac. Human Vacc Immunother 2012; 8:715–24. 10.4161/hv.19619 22495119

[25] Lee CH, Frasch CE Polysaccharide-protein conjugate vaccine. US patent number 8048432.

[26] Ravenscroft N, Hearshaw M, Martino A et al Physicalchemical and immunological characterization of the drug product (final lot) of a meningococcal group A conjugate vaccine [Abstract P101]. In: Abstracts of 16th International Pathogenic *Neisseria* Conference, 2008.

[27] Silveira IA, Bastos RC, Neto MS et al Characterization and immunogenicity of meningococcal group C conjugate vaccine prepared using hydrazide-activated tetanus toxoid. Vaccine 2007; 25:7261–70. 17719147 10.1016/j.vaccine.2007.07.037

[28] Bastos RC, de Carvalho JM, da Silveira IA, do Couto JS, Leandro KC Determination of hydrazine in a meningococcal C conjugate vaccine intermediary product. Vaccine 2010; 28:5648–51. 20598406 10.1016/j.vaccine.2010.05.079

[29] Gudlavalleti SK, Lee CH, Norris SE, Paul-Satyaseela M, Vann WF, Frasch CE Comparison of *Neisseria meningitidis* serogroup W polysaccharide-tetanus toxoid conjugate vaccines made by periodate activation of O-acetylated, non-O-acetylated and chemically de-O-acetylated polysaccharide. Vaccine 2007; 25:7972–80. 17936445 10.1016/j.vaccine.2007.06.018

[30] Kshirsagar N, Mur N, Thatte U et al Safety, immunogenicity, and antibody persistence of a new meningococcal group A conjugate vaccine in healthy Indian adults. Vaccine 2007; 25S:A101–7. 17532101 10.1016/j.vaccine.2007.04.050

